# A Novel Starfish Optimization Algorithm for Secure STAR-RIS Communications

**DOI:** 10.3390/biomimetics11040243

**Published:** 2026-04-03

**Authors:** Mona Gafar, Shahenda Sarhan, Abdullah M. Shaheen, Ahmed S. Alwakeel

**Affiliations:** 1 Department of Computer Engineering and Information, College of Engineering, Wadi Ad Dwaser, Prince Sattam Bin Abdulaziz University, Al-Kharj 16278, Saudi Arabia; m.gafar@psau.edu.sa; 2 Computer Science Department, Faculty of Computers and Information, Mansoura University, Mansoura 35516, Egypt; sh_sarhan@mans.edu.eg; 3 School of Computer Science and Technologies, VIZJA University, 01-043 Warsaw, Poland; 4 Department of Electrical Engineering, Faculty of Engineering, Suez University, Suez P.O. Box 43221, Egypt; abdullah.mohamed.eng19@suezuni.edu.eg

**Keywords:** reconfigurable intelligent surfaces, simultaneously transmitting and reflecting, achievable rate limitation, starfish optimizer

## Abstract

This paper develops an intelligent Enhanced Starfish Optimization (ESFO) algorithm for optimizing a secure wireless communication infrastructure. The Starfish Optimization (SFO) algorithm is inspired by starfish biology, using the integrated modeling of the arm-based exploration, preying, and regeneration behaviors of starfish. To further enhance the exploitation capability of the standard Starfish Optimization (SFO), the proposed Enhanced Starfish Optimization (ESFO) integrates a fitness-based interacting mechanism within the exploitation phase. This innovative modification improves local search accuracy, preserves population diversity, and mitigates premature convergence without introducing additional control parameters. Moreover, the proposed Enhanced Starfish Optimization (ESFO) is designed for secure wireless transmission, which is considered one of the main topics in next-generation wireless network infrastructure. The investigated network addresses the use of Simultaneously Transmitting and Reflecting RIS (STAR-RIS) in the security of the physical layer. This implemented STAR-RIS has a coupled phase shift to create reflected and transmission links, unlike traditional Reconfigurable Intelligent Surface (RIS). In this regard, we create a safe beamforming architecture that optimizes both Base Station (BS) precoding vectors and STAR-RIS transmission/reflection coefficients. In order to validate the efficiency of the proposed Enhanced Starfish Optimization (ESFO) algorithm, it is compared to several benchmark optimizers such as standard Starfish Optimization (SFO), Dhole Optimizer (DO), Neural Network Algorithm (NNA), Crocodile Ambush Optimization Algorithm (CAOA), and white shark Optimizer (WSO). These comparisons include several scenarios based on the transmitted power threshold which is varied in the range of 20 to 70 dBm with step of 5 dBm. The simulation results show that the proposed Enhanced Star Fish Optimization (ESFO) algorithm consistently outperforms existing benchmark approaches. This study supports future intelligent communication infrastructures in terms of secrecy and achievable rates over a range of transmit power levels. In particular, ESFO improves performance by up to 20–25% while converging 40–50% faster than traditional optimization algorithms, demonstrating its usefulness and resilience in STAR-RIS-assisted secure communication systems. The suggested ESFO-enabled architecture outperforms standard RIS-based systems in terms of secrecy capacity, according to numerical studies, and low-resolution STAR-RIS phase-shifters are sufficient to ensure robust secrecy performance.

## 1. Introduction

Security, throughput, latency, energy efficiency, deployment cost, hardware complexity, and end-to-end dependability have all had to be compromised in 5th-generation cellular network (5G) due to the rapid growth of Internet of Things (IoT) networks and the proliferation of smart devices [1,2,3]. In order to fully address these trade-offs and meet the demanding network requirements [4,5,6,7], sixth-generation cellular networks (6G) are being developed.

One of the deployment technologies for 6G is the use of RIS in wireless applications.

An RIS can assist a transmitter and receiver in establishing a Line-of-Sight (LOS) link using arrays of antenna elements [8,9,10], even in the presence of obstacles or insufficient power received from the straight path to create a stable connection.

Conventional reflecting-only RISs have limited application flexibility and electromagnetic control since they can only influence the wireless environment in the half-space facing the array. This inherent constraint severely limits coverage, user accessibility, and the geographic degrees of freedom available for system optimization. To overcome these limitations, the notion of STAR-RIS has recently emerged [11]. Unlike standard RISs, STAR-RISs can split the incident electromagnetic wave into transmission and reflection components, allowing for intelligent signal manipulation on both sides of the surface [12,13]. This dual feature enables STAR-RISs to build a genuinely full-space controlled radio environment, providing wider coverage, better design flexibility, and improved performance for next-generation wireless networks.

The intrinsic connection between the transmission and reflection coefficients of realistic STAR-RIS topologies makes ensuring safe transmission in STAR-RIS-enabled networks difficult. In full-space mutual eavesdropping, where both users and eavesdroppers receive signals from the same surface, this coupling makes it challenging to concurrently optimize the BS beamformers and STAR-RIS phases [14,15]. Consequently, the non-convex secrecy-rate maximization problem arising from linked phase constraints and multiuser fairness requirements is beyond the capabilities of conventional RIS optimization techniques [16,17]. In order to effectively utilize the STAR-RIS’s capacity to send and reflect signals concurrently, an effective optimization framework is required to prevent information loss.

Metaheuristic optimization techniques have gained popularity for handling highly non-convex and large-scale optimization problems in wireless communications, where traditional analytical methods are generally unfeasible. Among these approaches, the Starfish Optimization (SFO) algorithm has recently emerged as a bio-inspired population-based strategy that replicates starfish hunting, mobility, and regeneration behaviors. Because of its simple form and low implementation complexity, SFO may strike a balance between global exploration and local exploitation, making it appropriate for complicated engineering problems involving several coupled variables.

Despite these benefits, the typical SFO approach has a few drawbacks when used to severely limited communication system optimization situations. For instance, its exploitation phase may experience premature convergence in strongly coupled search spaces, and its inflexible interaction method may hinder adaptability to changing system conditions. These disadvantages become more obvious in STAR-RIS-assisted secure communication systems, where linked transmission–reflection coefficients, secrecy restrictions, and full-space signal propagation cause substantial interdependencies among optimization variables. As a result, there is a need to upgrade the conventional SFO framework with problem-aware procedures that improve convergence stability, robustness, and solution quality while minimizing algorithmic complexity.

Bio-inspired intelligence has evolved as an effective paradigm for addressing complicated, nonlinear, and high-dimensional optimization challenges in current wireless communication systems. Biomimetics, in particular, uses behavioral mechanisms found in natural creatures to develop adaptive and durable optimization algorithms. Motivated by this approach, the suggested work is inspired by a starfish’s biological properties, which include decentralized decision-making, adaptive foraging, and its regenerating ability in dynamic situations. These characteristics are particularly appropriate for modeling resource allocation and configuration issues in STAR-RIS-assisted networks, where several linked variables, stringent physical limits, and quickly changing channel conditions coexist.

In this regard, the proposed Enhanced Starfish Optimization algorithm (ESFO) converts fundamental biological behaviors of starfish into algorithmic operators that govern exploration and exploitation in the optimization process. The preying behavior is linked to global and local search processes that allow for efficient exploration of the solution space, whereas the regeneration and interaction methods reflect the starfish’s ability to recover from adverse conditions and adapt through collective behavior. By incorporating these biomimetic principles into the optimization of beamforming and coupled STAR-RIS coefficients, the proposed framework not only improves secrecy performance but also demonstrates how biologically inspired intelligence can effectively address emerging challenges in intelligent wireless communication systems, thus aligning the manuscript with the core scope of biomimetics research.

### 1.1. Related Work

A wide range of studies have looked into how RISs can increase achievable rates. The study in [18] investigated an adaptive clustering strategy for RIS installations in environments with blockages or weak propagation circumstances. To address such limitations, the authors proposed a self-organized RIS-assisted (SORA) system that improves transmission capacity by dynamically changing RIS behavior. However, when channels are significantly damaged, a single RIS may be insufficient to ensure consistent communication between the base station and user equipment. To solve this constraint, the authors presented a cluster-based RIS cooperation approach (C-RCA) in which multiple RIS units collaborate by progressively reflecting signals across clusters, increasing link robustness and overall system performance.

In another line of study, the authors of [19] concentrated on maximizing the achievable rate in uplink multiantenna IoT systems enabled with RISs. Their work optimized RIS phase settings, transmit power distribution, and BS beamforming in cases involving a large number of mobile IoT devices. Because these parameters are continuous and the objective is not concave, the joint optimization job was defined as a Markov decision process. A deep deterministic policy gradient (DDPG) reinforcement learning technique was then used to find the best control actions, followed by a safe action-shaping procedure to assure viable judgments.

Physical Layer Security (PLS) and RIS-assisted PLS in wireless communication systems have been the subject of various studies over the past few years (see [20,21,22,23,24,25] and the included references). For example, the authors of [20] concentrated on maximizing artificial noise power and RIS phase shifts in order to secure RIS-aided multiuser massive MIMO systems. In the meantime, ref.

[21] proposed virtual partitioning of RIS components to maximize secrecy capacity under rate restrictions and improve physical layer security. In order to confirm increased secrecy rates by RIS incorporation in the system, the authors of [22] jointly optimized transmit precoding, fake noise covariance, and RIS phase shifts. Additionally, the RIS phase-shift design and hybrid beamforming design were implemented in [23] to improve the system sum secrecy rate.

The authors of [24] used the Taylor series approximation to maximize secrecy rate and minimize power in a single-user/eavesdropper multiple-input multiple-output (MIMO) system. In order to maximize secrecy rates in single-user MIMO simultaneous wireless information and power transfer systems, an imprecise block coordinate descent method was used in [25]. Authors in [26] examined how to maximize the secrecy rate in single-cell multiple-input single-output (MISO) networks while taking the minimal collected energy constraint into account. In order to maximize secrecy throughput in wireless-powered communication networks, the primal decomposition method was applied [27].

On the other hand, RIS-assisted PLS systems have been studied in [28,29,30,31], especially under various imperfect CSI situations. For example, utilizing low-resolution programmable reflecting elements in an RIS for multiantenna access points serving single-antenna users amid multiple eavesdroppers, the authors of [28] suggested cooperative solutions for secure communications. In the meantime, ref. [29] presented a secure multicast communication system that uses RISs to thwart jammers and eavesdroppers during multiuser transmission. The application of active RISs to maximize worst-case secrecy rates and weighted sum secrecy rates under various imperfect CSI situations was examined in the analysis carried out in [30]. In [31], however, the authors presented an RIS-assisted MIMO secure communication system that maximizes ergodic secrecy rates through a joint optimization approach under statistical CSI and derivations based on random matrix theory.

In particular, the authors of [32] investigated a STAR-RIS-assisted MIMO network to optimize the weighted sum rate through an energy splitting (ES) scheme, and [33] developed customized schemes for the ES protocol and optimized training patterns for the time switching (TS) protocol to achieve effective uplink channel estimation in STAR-RIS-assisted two-user systems.

Since then, a number of studies have expanded STAR-RIS applications to include PLS approaches in secure communication systems.

In order to reduce full-space mutual eavesdropping, the authors of [34] presented a STAR-RIS-assisted secure communication system that optimizes coupled phase-shift coefficients using a penalty-based secrecy beamforming method. In a similar vein, ref. [35] investigated a number of transmission protocols, such as ES, mode switching (MS), and TS, and suggested coupled beamforming and transmission/reflection coefficient tuning to maximize the weighted sum secrecy rate. Additionally, ref. [36] used STAR-RIS to modify the electromagnetic environment, allowing genuine users and the BS to communicate securely while accounting for the full and statistical CSI of eavesdroppers. Although it lacked a robust beamforming architecture, ref. [37] assessed secrecy performance while taking residual hardware defects into account.

The Star Fish Optimization (SFO) algorithm is a recently developed bio-inspired metaheuristic optimization technique, first introduced by Zhong et al. [38]. Drawing from the natural behaviors of starfish, such as their exploration for food, preying mechanisms, and regenerative abilities, the SFO algorithm is presented to address complex global optimization problems in the search space. As a population-based algorithm, it simulates a group of starfish agents navigating the solution space to find optimal or near-optimal solutions.

The SFO algorithm is formulated by initializing a population of agents randomly within the search bounds, then updating positions based on fitness evaluations as it operates through two primary phases: exploration and exploitation, inspired by starfish ecology. In the exploration phase, agents employ a hybrid strategy combining multidimensional searches involving vector-based movements that mimic broad environmental scanning. The exploitation phase incorporates a two-directional search and special movement patterns, reflecting starfish preying and regeneration behaviors [39]. Preying captures prey from opposite directions while regeneration is adapted from suboptimal states. This enables focused refinement around promising areas, with regeneration mechanisms helping to escape stagnation [40].

As mentioned in the original SFO study, the technique was tested on ten real-world engineering optimization issues and outperformed numerous benchmark methods [38].

SFO and its variants have been applied to diverse fields, leveraging its balance of exploration and exploitation for complex, real-world optimization. In smart cities, the SFO algorithm was adopted for tuning the hyperparameters of deep learning frameworks (e.g., CNN-BiGRU-CrAM) and for detecting intrusions within IoT networks [41]. Also, a discrete SFO version was developed for solving the routing of the traveling salesman problem to achieve shorter tours in logistics and graph applications [42]. Additionally, an enhanced SFO variant was presented and applied for ultra-wideband indoor positioning to refine back propagation neural networks [43]. The SFO algorithm was utilized and integrated with machine learning for mine water identification models using Kolmogorov–Arnold networks, enhancing accuracy in laser-induced fluorescence data analysis [44]. Different successful implementations of the SFO were presented such as fuel cell parameter estimation [45], economic emission dispatch [46], virtual power plant optimization with mobile storage considering renewable energy uncertainty [47], and energy management in graph attention fusion networks [48]. These diverse applications underscore the SFO algorithm’s versatility, efficiency and applicability. However, research has quickly progressed to enhancements addressing limitations like slow convergence or local optima trapping [43].

### 1.2. Research Motivation

The emergence of STAR-RIS technology opens up new possibilities for constructing fully programmable wireless environments by allowing signals to be transmitted and reflected simultaneously across the space domain. However, the real implementation of STAR-RIS involves coupled phase-shift limitations, complicating system design and limiting the effectiveness of traditional optimization approaches. At the same time, providing secure communication has become more difficult due to the broadcast nature of wireless channels and the presence of full-space mutual eavesdropping. These characteristics necessitate innovative optimization frameworks capable of constructing transmit beamforming and STAR-RIS coefficients simultaneously while respecting the intrinsic coupling structure. Existing convex or learning-based approaches frequently struggle with the ensuing substantially non-convex and multidimensional search space, highlighting the need for an efficient, adaptable, and robust optimization strategy. Motivated by the limitations of the standard SFO exploitation strategy, particularly its heavy reliance on the global best solution and reduced population diversity during later iterations, this study proposes an ESFO algorithm.

### 1.3. Main Contributions

This paper extends the original SFO by making specific improvements to overcome its shortcomings when used in STAR-RIS-assisted secure communication optimization situations. To maximize secrecy performance, the suggested technique develops the BS beamforming vectors and STAR-RIS coefficients simultaneously under practical coupling limitations. The proposed ESFO introduces an adaptive, fitness-based interacting mechanism within the exploitation phase, allowing starfish to adjust their movements based on collective fitness information rather than solely following the best individual. By probabilistically combining the original two-directional preying behavior with interaction-based local intensification and stochastic peer perturbations, ESFO strengthens exploitation accuracy while maintaining sufficient diversity. This improvement improves robustness against premature convergence while maintaining the original SFO’s simplicity and computing efficiency without the need for additional control parameters. The main contributions of this work can be stated as follows:

- A new optimization framework for STAR-RIS-assisted secure communication is created, taking into consideration the coupled transmission–reflection phase-shift limitations.
- A novel ESFO is proposed to efficiently address the ensuing highly non-convex secrecy-rate maximization issue.
- To improve physical layer security under full-space mutual eavesdropping, BS beamforming and STAR-RIS coefficients are jointly optimized.
- Comprehensive performance comparisons with established benchmark algorithms (standard SFO [38], DO [49], NNA [50,51], CAOA [52], and WSO [53,54]) show that the ESFO produces consistently superior secrecy rates.
- Extensive simulations demonstrate the resilience, convergence behavior, and superiority of ESFO in coupled STAR-RIS systems with practical discrete phase resolutions.

The paper’s remaining sections are organized as follows: The system model is presented in Section 2. The construction of ESFO for an optimal coupled phase shift of STAR-RIS elements is described in Section 3. The simulation results to verify the effectiveness of the suggested strategy are included in Section 4. Section 5 concludes with a presentation of the study’s findings.

Notation: Matrices and column vectors are shown by bold uppercase and lowercase letters, respectively. The symbols $X^{-1}$, $X^{H}$, $X^{T}$, and $X^{†}$ stand for the inverse, conjugate transpose (Hermitian), transpose, and pseudo-inverse of a matrix X. The trace of matrix X is shown by the operator tr(X) and the Euclidean norm is represented by ∥·∥. A circularly symmetric complex Gaussian vector with mean μ and covariance matrix Γ is represented by a random vector x∼CN(μ,Γ). The set of all complex numbers is represented by the symbol C, whereas complex matrices and column vectors of dimensions N×M and N×1 are denoted by $C^{N\times M}$ and $C^{N\times 1}$, respectively. The M×M identity matrix is denoted by $I_{M}$.

## 2. System Model

### 2.1. Communication Scenario and Channel Setup

**Figure 1:**
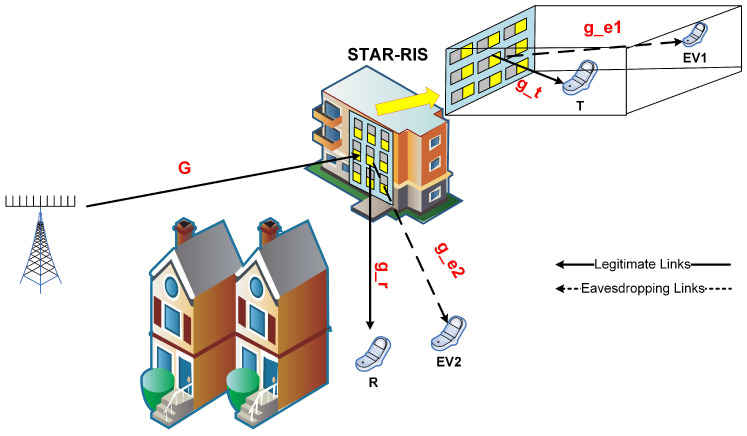
Proposed STAR-RIS model.

We consider a secure downlink communication system in which a multiantenna BS, equipped with $N_{\;b}$ antennas, serves two authorized single-antenna users. One user is positioned on the transmission side of a STAR-RIS, known as the T-user, and the other on the reflection side, referred to as the R-user. Two single-antenna eavesdroppers, ${\mathrm{Ev}}_{1}$ and ${\mathrm{Ev}}_{2}$, are situated near the T-user and R-user, respectively (as shown in Figure 1).

A STAR-RIS consisting of *L* passive elements is installed between the BS and the users to overcome obstruction and allow full-space signal modification. Due to barriers or extreme attenuation, the direct BS-to-user and BS-to-eavesdropper lines are presumed to be unavailable or negligible, so all communication is routed through the STAR-RIS. The BS-RIS channel is represented as $G\in C^{L\times N_{\;b}}$, while the RIS-receiver links are denoted as $g_{t},g_{r},g_{e_{1}},g_{e_{2}}\in C^{L\times 1}$, corresponding to the T-user, R-user, ${\mathrm{Ev}}_{1}$, and ${\mathrm{Ev}}_{2}$, respectively. All channels follow a Rayleigh fading model and the perfect channel state information is assumed for analytical tractability.

### 2.2. Modeling of STAR-RIS Coupled Coefficients

A STAR-RIS consists of passive electromagnetic elements designed to simultaneously transmit and reflect the incident signal, allowing for full-space wireless coverage. The impinging electromagnetic wave is divided into two components for each STAR-RIS element: transmitted and reflected. This exclusive capability distinguishes STAR-RIS from traditional reflecting-only RIS and provides additional design flexibility.

The transmission and reflection responses of the *ℓ*-th STAR-RIS element are defined by complex coefficients

$$\gamma_{\ell }^{\left(t\right)}=\sqrt{\rho_{\ell }^{\left(t\right)}}\;e^{j\psi_{\ell }^{\left(t\right)}},\;\gamma_{\ell }^{\left(r\right)}=\sqrt{\rho_{\ell }^{\left(r\right)}}\;e^{j\psi_{\ell }^{\left(r\right)}},$$

where $\rho_{\ell }^{\left(t\right)}$ and $\rho_{\ell }^{\left(r\right)}$ are the transmission and reflection amplitudes of *ℓ* element, respectively, whereas $\psi_{\ell }^{\left(t\right)}$ and $\psi_{\ell }^{\left(r\right)}$ identify the corresponding phase shifts introduced by the STAR-RIS element.

Due to practical STAR-RIS implementations being passive and lossless, the transmission and reflection coefficients are not mutually exclusive. Specifically, the total power spread by each element must be conserved, giving rise to the following energy-splitting constraint

(1) $$\rho_{\ell }^{\left(t\right)}+\rho_{\ell }^{\left(r\right)}=1,\;\ell =1:L.$$

Furthermore, electromagnetic boundary constraints dictate a deterministic phase connection between the transmitted and reflected components. As a result, the phase shifts are intrinsically related and must meet the following phase-coupling constraint

(2) $$\left|\psi_{\ell }^{\left(t\right)}-\psi_{\ell }^{\left(r\right)}\right|=\frac{\pi }{2}$$

This constraint reflects the fact that the transmitted and reflected waves have a fixed phase offset, which considerably limits the possible design space for STAR-RIS coefficients. The diagonal matrices indicate that the STAR-RIS’s collective transmission and reflection behaviors are

$$\Gamma_{t}=\mathrm{diag}\;\left(\gamma_{1}^{\left(t\right)},\ldots ,\gamma_{L}^{\left(t\right)}\right),\;\Gamma_{r}=\mathrm{diag}\;\left(\gamma_{1}^{\left(r\right)},\ldots ,\gamma_{L}^{\left(r\right)}\right),$$

which describe the combined amplitude and phase responses of all STAR-RIS elements for the transmission and reflection links, respectively. These matrices shape the end-to-end channels and have a direct impact on the system’s secrecy performance.

### 2.3. Signal Model

The BS delivers two confidential information-bearing symbols, $x_{t}$ and $x_{r}$, intended for the T-user and R-user, respectively. These symbols are linearly precoded using beamforming vectors $w_{t}$ and $w_{r}\in C^{N_{\;b}\times 1}$, where each symbol is assumed to have unit average power, i.e., $E\{|x_{t}{|}^{2}\}=E\{|x_{r}{|}^{2}\}=1$. The overlaid transmit signal emitted by the BS can thus be represented as

(3) $$s=w_{t}x_{t}+w_{r}x_{r},\;C^{N_{b}\times 1}$$

which is subject to the total transmit power constraint discussed below. The received signal at a generic receiver χ∈{t,r}, which represents either the T-user or the R-user, after passing via the BS–STAR-RIS channel and being reshaped by the STAR-RIS coefficients, is given by

(4) $$y_{\chi }=g_{\chi }^{H}\Gamma_{\chi }Gs+n_{\chi },C^{1\times 1}$$

where G stands for the BS-to-STAR-RIS channel, $g_{\chi }$ for the STAR-RIS-to-receiver channel, and $\Gamma_{\chi }$ for the STAR-RIS transmission or reflection matrix, depending on the receiver’s position. Additive white Gaussian noise with a zero mean and variance $\sigma^{2}$ is modeled by the term $n_{\chi }$. By interpreting the unwanted signal as interference, each authorized user decodes its intended message.

With respect to STAR-RIS’s full-space radiation feature, passive eavesdroppers are inevitably able to detect confidential signals. The received signal at eavesdropper $e_{k}$, k∈{1,2}, is expressed as

(5) $$y_{e_{k}}=g_{e_{k}}^{H}\Gamma_{\eta (k)}Gs+n_{e_{k}},$$

$g_{e_{k}}$ represents the channel between the STAR-RIS and the *k*-th eavesdropper and η(k)∈{t,r} specifies whether the eavesdropper is located on the transmission or reflection side of the STAR-RIS. Similarly to legal users, the noise term $n_{e_{k}}$ is treated as additive white Gaussian noise with variance $\sigma^{2}$.

The achievable rate for the authorized user χ can be written as follows using the signal expressions mentioned above

(6) $$R_{\chi }=\log_{2}\;\left(1+\frac{|g_{\chi }^{H}\Gamma_{\chi }Gw_{\chi }{|}^{2}}{|g_{\chi }^{H}\Gamma_{\chi }Gw_{\bar{\chi }}{|}^{2}+\sigma^{2}}\right),$$

where $\bar{\chi }$ represents the unwanted user’s index (χ∈{t,r} and $\bar{\chi }\in \left\{t,r,\bar{\chi }\neq \chi \right\}$). Similarly, the rate at which the message meant for user χ can be decoded by eavesdropper $e_{k}$ is given by

(7) $$R_{e_{k},\chi }=\log_{2}\;\left(1+\frac{|g_{e_{k}}^{H}\Gamma_{\chi }Gw_{\chi }{|}^{2}}{|g_{e_{k}}^{H}\Gamma_{\chi }Gw_{\bar{\chi }}{|}^{2}+\sigma^{2}}\right).$$

Lastly, the non-negative difference between the highest rate that eavesdroppers may achieve and the genuine user rate is the secrecy rate that user χ can achieve. This is written as

(8) $$C_{\chi }={\left[R_{\chi }-\max\left\{R_{e_{1},\chi },R_{e_{2},\chi }\right\}\right]}^{+}.$$

where ${\left[a\right]}^{+}=\max\left\{a,0\right\}$.

### 2.4. Secure Joint Optimization Framework

By simultaneously adjusting the BS beamforming vectors and the STAR-RIS transmission/reflection parameters, we aim to ensure secure connection for both users. Our goal is to maximize the two legitimate users’ minimal secrecy rates. The formulation of the optimization problem is

(9a) $$\begin{array}{cc} \max_{w_{t},w_{r},\Gamma_{t},\Gamma_{r}}\; & \min\left\{C_{t},\;C_{r}\right\} \end{array}$$

(9b) $$\begin{array}{cc} \;\;\;\;s.t.\;\; & ∥w_{t}{∥}^{2}+{∥w_{r}∥}^{2}\leq P_{\max}, \end{array}$$

(9c) $$\begin{array}{cc} \; & \;\;\;\;\;\;\rho_{\ell }^{\left(t\right)}+\rho_{\ell }^{\left(r\right)}=1,\;\forall \ell , \end{array}$$

(9d) $$\begin{array}{cc} \; & \;\;\;\;\;\;\;\left|\psi_{\ell }^{\left(t\right)}-\psi_{\ell }^{\left(r\right)}\right|=\frac{\pi }{2},\;\forall \ell , \end{array}$$

(9e) $$\begin{array}{cc} \; & \;\;\;\;\;\;\;\;\;\;\;\;0\leq \rho_{\ell }^{\left(t\right)},\rho_{\ell }^{\left(r\right)}\leq 1,\;\psi_{\ell }^{\left(t\right)},\psi_{\ell }^{\left(r\right)}\in \left[0,2\pi \right). \end{array}$$

where $P_{max}$ denotes the transmit power budget at the BS, and (9b) refers to the overall power consumption constraint at the BS, whereas (9c) and (9d) represent the amplitude and phase-shift coupling constraints at the STAR-RIS, respectively.

The coupled STAR-RIS constraints, multiuser secrecy requirements, and joint BS-RIS design make this optimization issue highly non-convex, requiring the application of sophisticated global optimization techniques such as the proposed ESFO.

## 3. Proposed ESFO

### 3.1. Standard SFO

The SFO algorithm, introduced by Zhong et al. in 2025, is a bio-inspired metaheuristic that simulates starfish behavior via exploration and exploitation phases [38]. The exploration phase features a hybrid strategy of multidimensional searches, while the exploitation phase employs two-directional searching and regeneration mechanisms to refine solutions [55].

#### 3.1.1. Step 1: Initialization

An initial population of starfish positions is generated $X\in C^{N_{SF}\times D}$ uniformly within the search space:

(10) $$X_{i,j}=l_{j}+r_{1}\times \left(u_{j}-l_{j}\right);\;\;\;i=1:N_{SF},j=1:D$$

where $X_{i,j}$ indicates the position of each starfish (*i*) considering the *D*-dimension while the population matrix of starfish positions is denoted by (*X*). Also, $r_{1}$ refers to a randomized value while the maximum and minimum bounds of each dimension *j* are symbolized by $l_{j}$ and $u_{j}$. Then, the fitness of each starfish is estimated as $f(X_{i})$ and the current best starfish ($X_{Bst}$) is extracted.

#### 3.1.2. Step 2: Exploration Phase

In this stage, a multidimensional search strategy is modeled by randomly selecting five dimensions representing the starfish’s five arms. Consequently, those dimensions are updated using a sine/cosine-based movement toward the global best:

(11) $$\begin{array}{c} Xnew_{i}=\left\{\begin{array}{cc} X_{i}+a_{1}\times \left(X_{Bst}-X_{i}\right)\times \cos\left(\theta \right), & \mathrm{if}\;\;r_{2}\leq 0.5\\ X_{i}+a_{1}\times \left(X_{Bst}-X_{i}\right)\times \sin\left(\theta \right), & \mathrm{Else} \end{array}\right. \end{array}$$

Here, $r_{2}$ denotes independent random variables uniformly distributed in the interval (0,1). $Xnew_{i}$ illustrates the new position of the starfish (*i*) that upgrades its old position ($Xnew_{i}$), and $a_{1}$ is the expansion coefficient controlling step magnitude while θ is the search angle controlling rotation based on the iteration ratio as follows:

(12) $$a_{1}=\pi \times \left(2r_{3}-1\right)$$

(13) $$\theta =\frac{\pi }{2}\times \left(1-\frac{t}{t_{MX}}\right)$$

where *t* and $t_{MX}$ are the current iteration number and its maximum count. $r_{3}$ denotes independent random variables uniformly distributed in the interval (0,1).

#### 3.1.3. Step 3: Exploitation Phase

In this phase, each starfish can follow preying or regeneration behavior. In the first behavior, starfish perform a parallel two-directional search where five distances are computed between the best starfish and five randomly selected starfish:

(14) $$Dist_{m}=X_{Bst}-X_{i};\;\;\;\;m=1,2,3,4,5\;\;\mathrm{and}\;\;i=rand_{i}\left(1,N_{SF}\right)$$

where $Dist_{m}$ is the distance vector between global best and selected starfish (*i*). After that, only two distances are randomly chosen and the starfish position is updated by moving toward these directions:

(15) $$Xnew_{i}=X_{i}+r_{4}\times Dist_{m1}+r_{5}\times Dist_{m2}$$

where $r_{4}$ and $r_{5}$ are randomized values. This preying behavior encourages intensified local search while maintaining stochastic diversity.

In terms of the regeneration behavior, the worst starfish undergoes a regeneration process:

(16) $$Xnew_{i}=X_{W}\times e^{\left(\frac{t-N_{SF}}{t_{MX}}\right)}$$

where $X_{W}$ indicates the worst starfish that has the highest objective. This simulates slow arm regeneration and introduces new genetic material into the population. This gradually pulls it toward the origin, enabling exploration of new areas.

In the SFO algorithm, the control between exploration and exploitation throughout the optimization process is implemented via a regulating parameter (GP). It represents the probability of activating the exploration phase, while 1-GP determines the likelihood of executing the exploitation. In this study, GP is set to 0.5, ensuring an equal probability of exploration and exploitation, which provides a balanced search behavior without introducing additional tuning complexity. This probabilistic control mechanism allows the SFO algorithm to dynamically alternate between global and local search modes, contributing to its robust convergence performance across different optimization problems.

#### 3.1.4. Step 4: Boundary Control

In this phase, corrective boundary rules are applied for the control variables to ensure thwy stay within bounds after exploitation updates as follows:

(17) $$\begin{array}{c} Xnew_{i}=\left\{\begin{array}{cc} Xnew_{i}, & \mathrm{if}\;\;Xnew_{i}\leq l_{i}\\ u_{i}, & \mathrm{if}\;\;Xnew_{i}\geq u_{i} \end{array}\right. \end{array}$$

then, the new fitness values are estimated for the new starfish positions, and the global best starfish is upgraded. Steps 2–4 are iterated multiple times until the process stops when the maximum number of iterations is reached. Algorithm 1 states the Pseudocode of the standard SFO.

### 3.2. Proposed Enhanced Version of SFO (ESFO)

In the typical SFO, exploitation is managed by a two-way preying mechanism as given in Equation (15), which is supplemented by a regeneration cycle performed to the lowest-performing person, as indicated in Equation (16). An enhanced exploitation framework is suggested for augmenting the typical SFO local search capacity. This change strengthens intensification, increases solution variation in attractive areas, and reduces the risk of early convergence by introducing an adaptable, fitness-based interacting method during the exploitation stage.

In the proposed ESFO, the exploitation phase is probabilistically divided into two complementary modes controlled by a uniformly distributed random variable. For each starfish, the algorithm selects between the standard preying behavior and a fitness-guided interaction mechanism:

(18) $$\begin{array}{c} Xnew_{i}=\left\{\begin{array}{cc} \mathrm{Apply}\;\mathrm{Equation}\;\left(16\right)\;\mathrm{via}\;\mathrm{standard}\;\mathrm{SFO}, & \mathrm{if}\;\;r_{6}\leq 0.5\\ \mathrm{Apply}\;\mathrm{proposed}\;\mathrm{fitness}-\mathrm{based}\;\mathrm{interaction}, & \mathrm{Else} \end{array}\right. \end{array}$$

Here, $r_{6}$ denotes a random variable uniformly distributed in the interval (0,1). The proposed fitness-based interaction mechanism adaptively adjusts the search direction of each solution according to its relative fitness with respect to the current population, thereby enhancing exploitation while avoiding premature convergence. This probabilistic branching introduces adaptive behavior without adding extra control parameters.
**Algorithm 1** Standard SFO.**Require:** 
Objective function f(·); population size $N_{\mathrm{SF}}$; problem dimension *D*; lower and upper bounds l,u; maximum number of iterations $t_{\max}$; exploration probability GP**Ensure:** 
Best solution $X_{\mathrm{Bst}}$ and best fitness value $f(X_{\mathrm{Bst}})$1:Generate the initial population of starfish positions using Equation (1).2:Evaluate the fitness of each starfish $f(X_{i})$3:Identify the current best starfish position $X_{Bst}$.4:**for** t=1 to $t_{MX}$ **do**5:    Generate a random number r∈(0,1)6:    **if** r<GP **then**                                    ▹ activate the exploration phase7:        **for** i=1 to $N_{\mathrm{SF}}$ **do**8:           Compute rotation angle θ using Equation (3)9:           Compute expansion coefficient $a_{1}$ using Equation (4)10:         Update selected dimensions of $X_{i}$ using Equation (2)11:        **end for**12:    **else**                                        ▹ activate the exploitation phase13:        **for** i=1 to $N_{\mathrm{SF}}$ **do**14:           Randomly select five distinct dimensions corresponding to the starfish arms15:           Compute distance vectors using Equation (5)16:           Randomly select two distance vectors17:           Update each starfish position using the preying rule given in Equation (6).18:           Identify the worst-performing starfish $X_{W}$19:           Apply regeneration process to $X_{W}$ using Equation (7)20:        **end for**21:    **end if**22:    Apply boundary constraints to all updated starfish positions using Equation (8).23:    Evaluate the fitness of the updated population.24:    Update the global best starfish $X_{Bst}$ if a better solution is found.25:**end for**26:**return** the best solution $X_{Bst}$ and its fitness value.

When the fitness-based interacting method is activated, a cumulative interaction vector $DX_{i}$ is constructed by aggregating the relative influence of all starfish in the population:

(19) $$DX_{i}=\sum_{j=1}^{N_{SF}}\left(X_{j}-\left(V_{C}\times X_{i}\right)\right)\times sign\left(f_{i}-f_{j}\right)$$

where $f_{i}$ and $f_{j}$ denote the fitness values of starfish *i* and *j*, sign(·) controls attraction toward better solutions and repulsion from inferior ones, and $V_{C}$ is a randomly generated interaction coefficient introducing stochastic perturbation as follows:

(20) $$V_{C}=round\left(1+r_{6}+r_{1}\right)$$

where $r_{6}$ and $r_{7}$ denote two random values inside range [0,1]. This formulation enables collective learning by allowing each starfish to adapt its movement direction based on the fitness landscape. Also, two update rules are employed using the interaction vector $DX_{i}$.

(21) $$\begin{array}{c} Xnew_{i}=\left\{\begin{array}{cc} X_{Bst}+\frac{\epsilon ⊙DX_{i}}{N_{SF}}, & \mathrm{if}\;\;r_{7}\leq 0.1\\ X_{Bst}+\epsilon ⊙(X_{R}-X_{M}), & \mathrm{Else} \end{array}\right. \end{array}$$

where $\epsilon \in {\left(0,1\right)}^{D}$ is a random vector; ⊙ denotes element-wise multiplication; and $X_{R}$ and $X_{M}$ are two random starfish solutions that are unequally selected.

The upper rule is the intensified local exploitation that is activated with a small probability of 10% while the bottom rule illustrates the peer-based stochastic perturbation with a higher probability of 90%.

As shown, the proposed modification transforms the exploitation phase of SFO into a hybrid intensification framework that combines directional convergence toward the global best, fitness-aware collective interactions, and stochastic peer-based perturbations. This design enhances exploitation accuracy while maintaining sufficient diversity, thereby improving convergence stability and reducing the likelihood of stagnation in local optima. Moreover, the enhancement does not introduce additional control parameters, preserving the simplicity and usability of the original SFO. The ESFO steps are shown in Algorithm 2.
**Algorithm 2** Proposed ESFO.**Require:** 
Objective function f(·); population size $N_{\mathrm{SF}}$; problem dimension *D*; lower and upper bounds l,u; maximum number of iterations $t_{MX}$; exploration probability GP**Ensure:** 
Best solution $X_{\mathrm{Bst}}$ and best fitness value $f(X_{\mathrm{Bst}})$1:Generate the initial population of starfish positions using Equation (1)2:Evaluate the fitness of each starfish $f(X_{i})$3:Identify the current best starfish position $X_{Bst}$4:**for** t=1 to $t_{MX}$ **do**5:    Generate a random number r∈(0,1)6:    **if** r<GP **then**                                    ▹ activate the exploration phase7:        **for** i=1 to $N_{\mathrm{SF}}$ **do**8:           Compute rotation angle θ using Equation (3)9:           Compute expansion coefficient $a_{1}$ using Equation (4)10:         Update selected dimensions of $X_{i}$ using the sine/cosine-based exploration rule in Equation (2)11:        **end for**12:    **else**                                      ▹ activate the exploitation phase (ESFO)13:        **for** i=1 to $N_{\mathrm{SF}}$ **do**14:           Generate a random number $r_{e}\in \left(0,1\right)$15:           **if** $r_{e}>0.5$ **then**                                     ▹ standard SFO exploitation16:               Randomly select five distinct dimensions corresponding to the starfish arms17:               Compute distance vectors between the global best and the selected starfish using Equation (5)18:               Randomly select two distance vectors19:               Update each starfish position using the two-directional preying rule given in Equation (6)20:           **else**                                   ▹ fitness-guided interaction mechanism21:               Compute the interaction coefficient VC using Equation (11)22:               Compute the cumulative interaction vector ${\mathrm{DX}}_{i}$ using Equation (10)23:               Generate a random number $r_{7}\in \left(0,1\right)$24:               Update the starfish position using the enhanced exploitation rule in Equation (12)25:           **end if**26:           Identify the worst-performing starfish $X_{W}$27:           Apply regeneration process to $X_{W}$ using Equation (7)28:        **end for**29:    **end if**30:    Apply boundary constraints to all updated starfish positions using Equation (8)31:    Evaluate the fitness of the updated population32:    Update the global best starfish $X_{Bst}$ if a better solution is found33:**end for**34:**return** the best solution $X_{Bst}$ and its fitness value

Table 1 compares the structural differences between the original SFO and proposed ESFO. As demonstrated, the proposed ESFO’s update rule incorporates two complementary components: (i) a bounded stochastic exploration term responsible for maintaining diversity in early stages; (ii) a fitness-weighted attraction term that strengthens directional movement toward high-secrecy configurations; and (iii) adaptive control parameters that dynamically adjust the balance between these components as iterations progress. This hierarchical transition mechanism explains the enhanced convergence stability and statistically superior confidence intervals over the original SFO.

## 4. Numerical Results

This section presents numerical simulations to assess the performance of the proposed ESFO method. Figure 1 shows a three-dimensional Cartesian coordinate system with a BS at (0, 0, 0) m and a STAR-RIS with *L* passive elements deployed at (50, 0, 0) m. The intended users on the transmission and reflection sides are positioned at (50, 5, 0) m and (50, −5, 0) m, respectively, whereas the two eavesdroppers ${\mathrm{Ev}}_{1}$ and ${\mathrm{Ev}}_{2}$ are located at (50, 10, 0) m and (50, −10, 0) m.

The channel modeling takes into account both small-scale fading effects and large-scale path loss. In particular, the BS–STAR-RIS channel G and the STAR-RIS–receiver channels $g_{t}$, $g_{r}$, $g_{e_{1}}$, and $g_{e_{2}}$ are modeled as Rayleigh fading channels with distance-dependent attenuation. They can be written as $h=\sqrt{L_{0}d^{-\alpha }}\;\tilde{h}$, where $\tilde{h}$ represents the small-scale fading component with independent and identically distributed complex Gaussian entries with zero mean and unit variance. In this case, *d* is the matching link distance, α is the path-loss exponent, and $L_{0}$ is the reference path loss at a unit distance. The reference path loss in the simulations is set to $L_{0}=-30$ dB, the BS–STAR-RIS link’s path-loss exponent is selected as $\alpha_{\mathrm{BS}}=2.2$, and the STAR-RIS-to-user and STAR-RIS-to-eavesdropper links are defined by $\alpha_{T,S}=\alpha_{R,S}=\alpha_{E_{1},S}=\alpha_{E_{2},S}=2.5$. The convergence threshold is set to $\varepsilon_{\mathrm{th}}=10^{-3}$, the noise power is fixed at $\sigma^{2}=-105$ dBm, and the algorithm-related parameters are chosen as $c_{1}=c_{2}=0.99$. Additionally, to guarantee statistically reliable performance evaluation, all numerical results are averaged over 100 independent realizations.

To evaluate the effectiveness of the proposed ESFO algorithm, simulation results are divided into four parts. First, a statistical comparison is performed by calculating the mean, maximum, minimum, and standard deviation of the objective values obtained by the proposed technique and numerous benchmark algorithms. Second, box plots and histograms are used to provide a thorough visual representation of the performance distribution, emphasizing the proposed algorithm’s robustness and superiority. Third, convergence curves are shown to illustrate convergence behavior and show that the suggested methodology has a faster convergence speed than competing methods. Finally, the suggested ESFO algorithm achieves significant performance increases, as measured by its achievable rate performance.

All simulations were carried out using MATLAB R2022a. The experiments were executed on the a workstation with an Intel Core i7 CPU (3.6 GHz) and 16 GB RAM, running Windows 11 (64-bit). All algorithms were developed in identical software and hardware conditions to ensure fair computational comparison and reproducibility of the reported findings.

### 4.1. Statistical Outcomes

Table 2 shows the statistical findings of the proposed ESFO method versus CAOA, DO, NNA, standard SFO, and WSO under various transmit power constraints $P_{\max}$. It has been observed that ESFO consistently delivers the highest mean secrecy performance, with relative improvements of up to several tens of percent compared to the benchmark algorithms at all power constraint levels. In particular, when compared to CAOA and DO, ESFO gives an average performance improvement of 15–30%, outperforming NNA and WSO by 10–25%, demonstrating its superior optimization capabilities. Compared to the standard SFO, the suggested approach results in an extra 8–15% improvement, demonstrating the efficacy of the proposed fitness-guided interaction and adaptive exploitation mechanisms. Furthermore, ESFO’s minimum secrecy values are much greater than those of competing approaches, suggesting increased robustness and reduced performance deterioration, while the maximum values show that ESFO is capable of achieving superior global solutions. Although a slight rise in standard deviation is observed at higher power levels, this behavior reflects ESFO’s improved exploration ability, which effectively prevents premature convergence.

**Figure 2:**
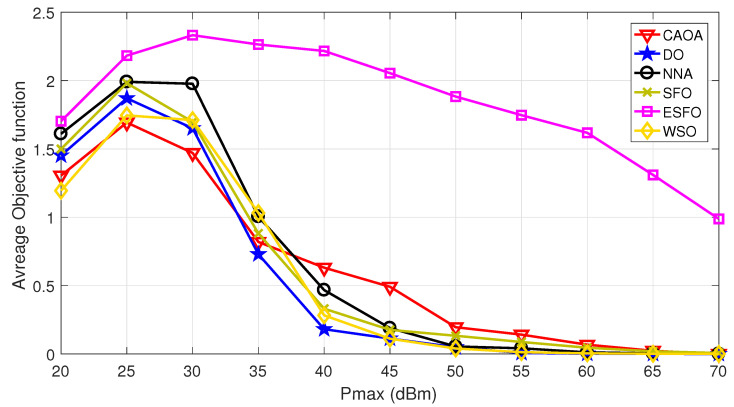
Average objective function for (CAOA, DO, NNA, SFO, ESFO, and WSO) optimization algorithms at different power thresholds.

Figure 2 illustrates the average objective function values for CAOA, DO, NNA, standard SFO, ESFO, and WSO at various transmit power levels ($P_{\max}=\left[20:70\right]$ dBm). As power constraints grow, all algorithms begin to increase in their objective values. However, performance changes at $P_{\max}=30$ dBm as the objective value decreases. This occurs because as the power constraint is increased, the achievable rate of intended users and eavesdroppers grows as well. The suggested ESFO consistently outperforms all existing algorithms in average objective values across the whole power range. In particular, ESFO provides an average improvement of 10–20% over CAOA and DO, 8–15% over NNA and WSO, and 5–10% over standard SFO at medium and high power levels. These relative improvements become more evident as transmit power increases, proving ESFO’s effectiveness in utilizing the expanded possible area and verifying its better optimization capabilities in STAR-RIS-assisted secure communication systems.

### 4.2. Performance Analysis

**Figure 3:**
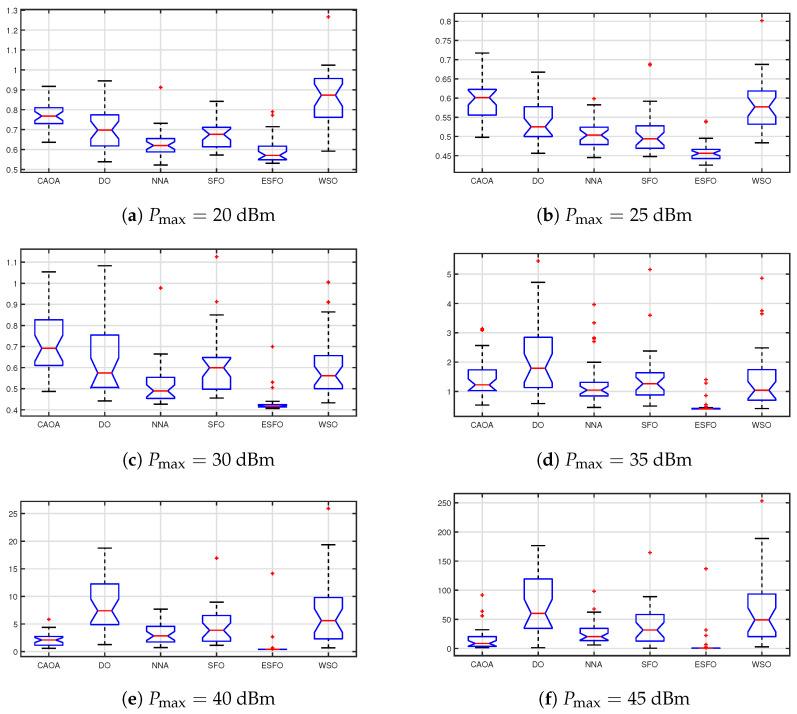
Box plot obtained by CAOA, DO, NNA, SFO, ESFO, and WSO over 100 runs.

Figure 3 depicts box plots of the objective function values acquired by CAOA, DO, NNA, standard SFO, ESFO, and WSO across 100 independent runs. The suggested ESFO algorithm has the greatest median value of any approach, but its interquartile range is reduced by around 20–35% when compared to CAOA, DO, and NNA, showing increased stability. Furthermore, the spread between the minimum and maximum values of ESFO is significantly narrower than that of the benchmark algorithms, indicating greater resistance to random initialization.

**Figure 4:**
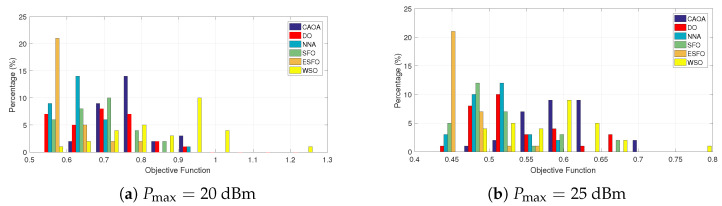

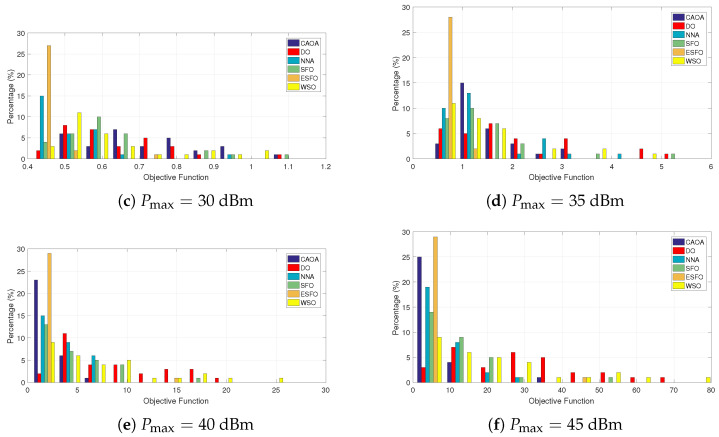
Histogram obtained by CAOA, DO, NNA, SFO, ESFO, and WSO over 100 runs.

Figure 4 presents the histogram distributions of the objective function values acquired by CAOA, DO, NNA, standard SFO, ESFO, and WSO across 100 independent runs for a range of power constraints (20 to 45 dBm). Results indicate that the proposed ESFO algorithm generates a highly concentrated distribution around higher objective values, with more than 65–75% of runs falling within the top performance range. In contrast, the benchmark algorithms have broader distributions, with a major fraction of their outputs pushed toward lower goal values. Specifically, ESFO raises the likelihood of achieving high-quality solutions by 20–30% when compared to CAOA and DO, and by 10–20% when compared to NNA, SFO, and WSO. This tighter distribution and increased occurrence of near-optimal solutions illustrate ESFO’s durability and reliability, demonstrating its exceptional ability to regularly achieve high-performance solutions in STAR-RIS-assisted secure communication optimization issues.

### 4.3. Comparative Convergence Analysis

**Figure 5:**
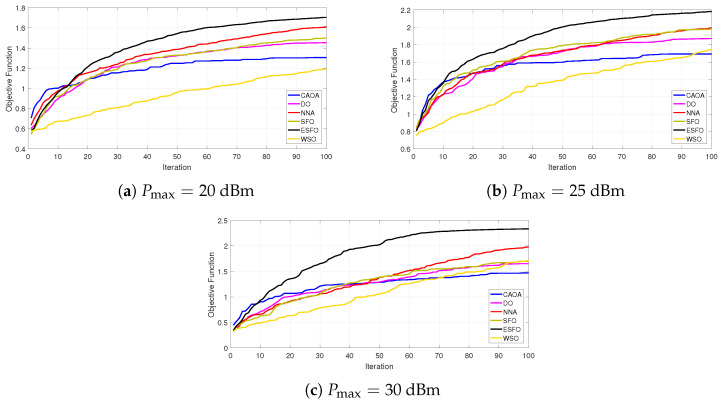
Average convergence rates of different optimization algorithms (proposed ESFO, standard SFO, DO, CAOA, NNA, and WSO).

Figure 5 compares the average convergence behavior of the proposed ESFO technique to standard SFO, DO, CAOA, NNA, and WSO. All algorithms show rapid improvement during the initial iterations; however, ESFO converges faster and achieves a higher goal value with fewer iterations. In particular, ESFO achieves near-stable convergence in around 40–50% fewer iterations than CAOA and DO, and 25–35% fewer iterations than NNA and WSO. Compared to the traditional SFO, the suggested modifications allow ESFO to accelerate convergence by about 15–20% while avoiding premature stagnation. These findings demonstrate that the adaptive exploitation and fitness-guided interaction mechanisms built into ESFO successfully balance exploration and exploitation, resulting in faster and more consistent convergence in STAR-RIS-assisted secure communication optimization.

### 4.4. Achievable Rates

Table 3 compares the proposed ESFO algorithm’s achievable rates to those reached by CAOA, DO, NNA, standard SFO, and WSO at various transmit power thresholds $P_{\max}=\left[20,30,50\right]$ dBm. It shows the achievable secrecy rates for legitimate T-users and R-users, indicating the most reliable transmission rates while maintaining confidentiality from prospective eavesdroppers. As expected, the achievable rates of all algorithms rise with available transmit power as signal strength improves and the feasible solution space expands. Nonetheless, ESFO regularly obtains the highest possible rates at all power levels. At $P_{\max}=20$ dBm, ESFO increases the achievable rate by around 8–15% compared to CAOA and DO, and by about 5–10% compared to NNA and WSO. When the power threshold is raised to $P_{\max}=30$ dBm, the performance advantage of ESFO becomes more obvious, reaching 12–20% over CAOA and DO, and roughly 8–12% over standard SFO. At high transmit power ($P_{\max}=50$ dBm), ESFO maintains a distinct performance advantage, achieving up to 20–25% higher attainable rates than the weakest-performing benchmarks while beating regular SFO by 10–15% on average. These findings show that the proposed ESFO algorithm successfully utilizes higher power resources and provides significant performance improvements in STAR-RIS-assisted secure communication systems.

### 4.5. Robustness, Variability and Confidence Interval Analysis

The robustness of the compared algorithms was evaluated. Also, two recent algorithms (ECO, SSO) were additionally implemented in this evaluation. The Educational Competition Optimizer (ECO) is a recently introduced metaheuristic algorithm proposed in 2024 by J. Lian et al. [56]. It is conceptually inspired by the hierarchical and competitive dynamics observed across different educational stages—namely elementary, middle, and high school levels. ECO models the progressive intensification of competition, where the number of participants decreases while performance pressure increases, by structuring the optimization process into three sequential phases. This staged mechanism enables a balance between exploration and exploitation throughout the search process. Owing to its robust search capability, ECO has been successfully deployed in a variety of engineering applications, including constrained optimization problems [57,58], coordination of overcurrent relays in renewable-based microgrids [59], parameter estimation in fuel cells [60], and frequency regulation in PV-integrated power systems [61].

In parallel, the Somersaulting Spider Optimizer (SSO), introduced in 2025, represents a novel class of bio-inspired metaheuristics derived from the unique locomotion behavior of the somersaulting or flic-flac spider. This desert-adapted species exhibits the two distinct movement strategies of a rapid somersaulting motion for escape and a slower rolling motion for exploration and foraging. These strategies were mathematically abstracted within SSO to balance global exploration and local exploitation. It has demonstrated promising performance and has been successfully hybridized with machine learning techniques, particularly in enhancing predictive modeling accuracy for applications such as concrete compressive strength estimation using advanced regression models [62].

For CAOA, DO, SFO, NNA, WSO, ECO, SSO, and ESFO, the variance and coefficient of variation (CV) are reported, as shown in Table 4. As reported, ESFO clearly outperforms all competing methods in terms of robustness. At a $P_{\max}$ of 30 dBm, the ESFO achieves the lowest variance ($4.48\times 10^{-2}$) among all algorithms. Compared to CAOA, DO, SFO, NNA, and WSO, ESFO reduces variance by approximately 56.1%, 61.2%, 58.6%, 43.7%, and 69.3%, respectively. These substantial reductions confirm that ESFO exhibits significantly less dispersion in its obtained solutions, reflecting superior convergence stability.

Similarly, ESFO records the lowest coefficient of variation (CV = 0.0908), indicating the strongest robustness relative to its mean performance. In quantitative terms, ESFO improves CV by 58.2% compared to CAOA, 55.9% relative to DO, 36.4% compared to NNA, 53.1% against SFO, and 59.3% compared with WSO. This demonstrates that ESFO not only produces higher-quality solutions but does so with markedly improved consistency across repeated executions.

Beyond robustness, the 95% bootstrap confidence intervals (CIs) of the mean objective values further highlight the superiority of ESFO in this maximization problem. ESFO achieves the highest mean objective range, with a confidence interval of [2.2465, 2.3953], which is entirely separated from the intervals of all other algorithms. The lower bound of ESFO alone exceeds the upper bounds of CAOA, DO, SFO, and WSO, confirming statistically and practically superior performance.

Similar findings are attained at other threshold powers of 35, 40 and 45 dBm. Therefore, these results demonstrate that ESFO achieves the best trade-off between solution quality and robustness, delivering not only the highest objective values but also the most stable and repeatable performance among all compared algorithms. This robustness advantage is particularly important for real-world optimization problems, where consistency across runs is as critical as achieving high objective values.

### 4.6. Hypothesis Analysis

In order to conduct hypothesis testing, the Wilcoxon signed-rank test results across several threshold power ($P_{\max}$ of 30, 35, 40 and 45 dBm) scenarios are implemented, as shown in Table 5. The results confirm the statistical superiority of ESFO in most operating conditions. For a $P_{\max}$ of 30 and 35 dBm, ESFO demonstrates consistent and statistically significant improvements over all competing algorithms, with extremely small *p*-values (≤9.71 $\times 10^{-5}$), indicating strong dominance and reliable convergence behavior.

At a $P_{\max}$ of 40 dBm, ESFO maintains statistically significant superiority over DO, NNA, SFO, and WSO. However, the comparison with CAOA becomes statistically insignificant (p=0.147), suggesting comparable performance between ESFO and CAOA under this specific operating condition. This indicates that CAOA temporarily narrows the performance gap at intermediate power limits. When $P_{\max}$ increases to 45 dBm, ESFO regains clear and statistically significant dominance over all algorithms, reaffirming its robustness and scalability under higher operating constraints. The consistently high signed-rank values in ESFO vs. WSO comparisons further highlight the strong and systematic advantage of ESFO. Thus, these results demonstrate that ESFO delivers stable, statistically significant performance gains across varying system capacities, confirming its effectiveness and robustness under different operational scenarios.

### 4.7. Computational Complexity and Runtime Analysis

The “Big O notation,” a widely used mathematical notation used in computer sciences [63], could be utilized to do a computational complexity analysis of metaheuristic methods. To begin, $N_{SF}$ represents the total number of solutions per iteration, while $t_{MX}$ is the maximum iteration count. The first phase representing the exploration feature simulates either the multidimensional searching or vector-based movements of the agent. Addressing this phase, the computational complexity is $O(t_{MX}\times \frac{N_{SF}}{2}\times f\left(x\right))$, where f(x) is the evaluation of the fitness function. The second phase regarding exploitation incorporates either the two-directional search or special movement patterns. The complexity part in computing this phase is $O(t_{MX}\times \frac{N_{SF}}{2}\times f\left(x\right))$. Consequentially, the total computational complexity records $O(t_{MX}\times N_{SF}\times f\left(x\right))$. The suggested ESFO has comparable computational complexity to the other methods used (standard SFO, CAOA, DO, NNA, and WSO) in terms of Big O notation. The computational cost of these techniques for our RIS issue is O(2000), with a population size of 20 and 100 iterations.

To supplement the theoretical analysis, actual execution times and memory usage were measured in MATLAB using the same hardware and software environments. The results are summarized in Table 6.

Table 6 displays the elapsed runtime in seconds and MATLAB memory consumption in MB for each compared algorithm. The proposed ESFO has an elapsed time of 1.3842 s, which is less than the majority of the algorithms used, including SFO, DO, CAOA, and NNA, and just slightly more than WSO, exhibiting competitive computational efficiency. In terms of memory consumption, all algorithms have similar levels (about 3298–3353 MB), with ESFO consuming 3353 MB, which is still within the same computational scale as the other approaches.

The improved resilience metrics given in the experimental section are due to the modified interaction dynamics of ESFO. First, the fitness-based interaction minimizes random drift in high-dimensional space. Second, the adaptive exploration technique reduces premature convergence. Third, a controlled search focus on high-quality regions helps to stabilize late-stage refining.

These approaches work together to reduce dispersion in final solutions over independent runs, yielding lower variance, smaller coefficients of variation, and persistent statistical dominance. As a result, the statistical improvements are more than just descriptive outcomes; they reflect structural behavioral changes in the search dynamics of the proposed ESFO algorithm.

### 4.8. Benchmark Validation and Scalability Analysis

To validate the ESFO and SFO’s efficiency, they are applied to three benchmark issues linked to the CEC 2017 competition [64]. The three functions are: (F1) shifted and rotated Rastrigins, (F2) shifted and rotated noncontinuous Rastrigins, and (F3) hybrid function 1 (N=1). They are evaluated with multiple dimensions of 2, 10, and 30 to investigate scalability, with each dimension’s bounds ranging from −100 to 100.

For each benchmark, 50 different runs are performed using both the proposed ESFO and the standard SFO. Table 7 shows the best, average, worst, and standard deviations. Table 7 shows that the suggested ESFO outperforms the original SFO in terms of efficacy and scalability. In the low-dimensional scenario (D=2), both algorithms attain identical optimal values for all benchmark functions (F1–F3) with zero standard deviation, demonstrating that both methods can identify the global optimum in simple search spaces.

However, as the dimensionality increases, the superiority of ESFO becomes more apparent. For D=10, ESFO consistently produces lower best, average, and worst values in all three functions. For example, in F1, average fitness rises from 520.4874 (SFO) to 516.4368 (ESFO), while standard deviation falls from 5.8615 to 5.1179, indicating greater robustness. Similarly, the average value of F2 is dropped from 822.7479 to 820.6951 and F3 from 1104.9692 to 1104.9072. Although the gains at D=10 are minor, they continuously favor ESFO in terms of solution quality and stability.

The performance disparity widens dramatically at D=30, illustrating ESFO’s scalability advantage. In F1, the average fitness value reduces significantly from 725.9997 (SFO) to 650.8450 (ESFO), reflecting an improvement of roughly 10.35%. Similarly, for F2, the average value improves from 1022.9398 to 939.4327 (about 8.17%), and for F3, from 1317.6335 to 1262.9772 (about 4.15%). Furthermore, ESFO typically obtains lower worst-case values and lower standard deviations, particularly in F2 and F3, where the dispersion reduction suggests greater convergence stability in high-dimensional and complex environments.

## 5. Conclusions

This paper presents an enhanced variant of the Starfish Optimization (SFO) algorithm designed to improve the exploitation capability of the standard SFO while preserving its inherent simplicity and low parameter dependency. The modified exploitation framework enhances starfish movements by utilizing collective fitness information, improving local search accuracy and minimizing premature convergence, all without adding control parameters. This maintains the computational efficiency of the original SFO. The integration of preying behavior with the interaction-based strategy creates a hybrid mechanism that is robust and adaptable to various optimization landscapes. The obtained results demonstrate that Enhanced Starfish Optimization (ESFO) provides superior convergence behavior and solution quality compared to the standard SFO, particularly for complex, nonlinear, and multimodal optimization problems. These findings highlight the effectiveness of the proposed enhancement and confirm the potential of ESFO as a competitive and reliable optimization tool. Future work may extend ESFO to constrained and multi-objective optimization problems, as well as explore adaptive control schemes and hybridization with problem-specific heuristics to further improve performance in large-scale and real-world applications.

## Figures and Tables

**Table 1:** Structural differences between original SFO and proposed ESFO.

Aspect	Original SFO	Proposed ESFO
Exploration Strategy	Fixed stochastic exploration behavior	Adaptive exploration with iteration-dependent regulation
Exploitation Mechanism	Standard attraction toward elite solutions	Fitness-guided weighted attraction with adaptive intensity
Exploration–Exploitation Balance	Implicit and static balance	Explicit and dynamically controlled transition mechanism
Behavior Under Strong Variable Coupling	May stagnate in tightly coupled regions	Enhanced directional pressure mitigates stagnation
Population Contraction	Natural convergence without structured control	Controlled contraction improving stability and robustness
Observed Performance Impact	Moderate variance across runs	Reduced variance, narrower confidence intervals, and improved robustness

**Table 2:** Statistical outcomes of the proposed ESFO against other benchmark algorithms (CAOA, DO, NNA, standard SFO, and WSO).

		Optimization Algorithms					
		CAOA	DO	NNA	SFO	ESFO	WSO
$P_{\max}=$ 20 dBm	**Min**	1.089902	1.058037	1.096627	1.187977	**1.265813**	0.789727603
	**Max**	1.570849	1.85717	1.913915	1.747525	**1.884383**	1.68924829
	**Mean**	1.30491	1.452538	1.610647	1.499885	**1.703137**	1.194636865
	**STD**	**0.117044**	0.206934	0.164145	0.173525	0.171763	0.200845882
$P_{\max}=$ 25 dBm	**Min**	1.394269	1.498022	1.670946	1.451811	**1.85234**	1.24717459
	**Max**	2.008952	2.192469	2.245581	2.234114	**2.351294**	2.068451958
	**Mean**	1.693341	1.870556	1.990701	1.981269	**2.182726**	1.745447764
	**STD**	0.14447	0.195398	0.144783	0.204385	**0.118286**	0.191156736
$P_{\max}=$ 30 dBm	**Min**	0.948842	0.923045	1.022966	0.888375	**1.429356**	0.994425067
	**Max**	2.052012	2.260676	2.342536	2.194333	**2.459479**	2.308712147
	**Mean**	1.470461	1.651021	1.976948	1.697755	**2.33236**	1.711466863
	**STD**	0.319398	0.339844	0.282074	0.329021	**0.21171**	0.381960673
$P_{\max}=$ 35 dBm	**Min**	0.31864	0.183501	0.252311	0.193796	**0.712136**	0.205713244
	**Max**	1.867027	1.71079	2.21021	1.997787	**2.497658**	2.408904577
	**Mean**	0.819554	0.7297	1.005026	0.881915	**2.264326**	1.031505042
	**STD**	**0.351906**	0.478196	0.512157	0.40977	0.493143	0.575481151
$P_{\max}=$ 40 dBm	**Min**	**0.171214**	0.053358	0.129765	0.059079	0.070692	0.038570288
	**Max**	1.681878	0.794573	1.367447	0.869514	**2.497805**	1.451835824
	**Mean**	0.631351	0.181195	0.467437	0.331345	**2.216907**	0.282714334
	**STD**	0.372174	**0.156483**	0.346336	0.212323	0.599251	0.285885354
$P_{\max}=$ 45 dBm	**Min**	0.028011	0.014268	**0.034004**	0.01909	0.022918	0.0124168
	**Max**	1.483732	1.886491	0.483784	0.795286	**2.499304**	0.819430151
	**Mean**	0.491753	0.112831	0.189441	0.176372	**2.054593**	0.11186297
	**STD**	0.387344	0.336812	**0.128704**	0.190784	0.777581	0.150910018
$P_{\max}=$ 50 dBm	**Min**	**0.010907**	0.005664	0.010183	0.006079	0.007306	0.003949056
	**Max**	0.815888	0.811119	0.166579	2.161922	**2.499752**	0.351601263
	**Mean**	0.195687	0.049277	0.054993	0.133246	**1.883123**	0.039873808
	**STD**	0.208243	0.145241	**0.036912**	0.392651	0.892815	0.063615415

**Table 3:** Achievable rates of the proposed ESFO against other benchmark algorithms (CAOA, DO, NNA, standard SFO, and WSO) for different power thresholds ($P_{\max}=\left[20,30,50\right]$ dBm).

		Optimization Algorithms					
		CAOA	DO	NNA	SFO	ESFO	WSO
$P_{\max}=$ 20 dBm	$R_{t}$	2.407398	3.100565	3.267821	2.772627	3.141149	2.5687512
	$R_{e1,t}$	0.836549	1.243396	1.343659	1.025101	1.253257	0.912635647
	$R_{e2,t}$	0.836549	1.243396	1.343659	1.025101	1.253257	0.912635647
	$R_{r}$	2.997694	3.101449	3.225045	3.167745	3.148297	2.690310913
	$R_{e1,r}$	1.24417	1.234138	1.31113	1.35982	1.263913	1.023797266
	$R_{e2,r}$	1.24417	1.234138	1.31113	1.35982	1.263913	1.023797266
	$C_{t}$	1.570849	1.85717	1.924162	1.747525	1.887892	1.656115553
	$C_{r}$	1.753524	1.867311	1.913915	1.807925	1.884383	1.666513646
$P_{\max}=$ 30 dBm	$R_{t}$	5.413586	7.356727	5.96208	6.662309	7.835573	5.178353681
	$R_{e1,t}$	3.361574	4.92006	3.619543	4.399209	5.367331	2.958800406
	$R_{e2,t}$	3.361574	4.92006	3.619543	4.399209	5.367331	2.958800406
	$R_{r}$	4.219486	4.818414	8.236069	4.858796	7.370266	4.611254449
	$R_{e1,r}$	2.066548	2.557738	5.824391	2.664463	4.910787	2.397967649
	$R_{e2,r}$	2.066548	2.557738	5.824391	2.664463	4.910787	2.397967649
	$C_{t}$	2.052012	2.436667	2.342536	2.263101	2.468241	2.219553274
	$C_{r}$	2.152938	2.260676	2.411679	2.194333	2.459479	2.2132868
$P_{\max}=$ 50 dBm	$R_{t}$	9.812048	11.55328	13.7864	10.12222	18.06932	9.587738121
	$R_{e1,t}$	8.996161	10.50747	13.1327	7.631665	15.56948	9.383933432
	$R_{e2,t}$	8.996161	10.50747	13.1327	7.631665	15.56948	9.383933432
	$R_{r}$	11.11503	12.60914	7.346617	16.22158	14.89364	6.82381551
	$R_{e1,r}$	10.17983	11.79802	7.180038	14.05966	12.39389	6.762619653
	$R_{e2,r}$	10.17983	11.79802	7.180038	14.05966	12.39389	6.762619653
	$C_{t}$	0.815888	1.045801	0.653703	2.490559	2.499842	0.203804689
	$C_{r}$	0.935201	0.811119	0.166579	2.161922	2.499752	0.061195857

**Table 4:** Robustness, variability analysis and mean objective values with 95% confidence intervals.

$P_{\max}$ **= 30 dBm**			
**Algorithm**	**Variance**	**CV**	**95% CI (Mean Objective)**
**CAOA**	$1.020152\times 10^{-1}$	0.2172	[1.359380, 1.584173]
**NNA**	$7.956571\times 10^{-2}$	0.1427	[1.871415, 2.070773]
**ESFO**	$4.482115\times 10^{-2}$	0.0908	[2.246508, 2.395268]
**SFO**	$1.082551\times 10^{-1}$	0.1938	[1.580677, 1.809525]
**DO**	$1.154941\times 10^{-1}$	0.2058	[1.531005, 1.770250]
**WSO**	$1.458940\times 10^{-1}$	0.2232	[1.569907, 1.843350]
**ECO**	$8.330408\times 10^{-2}$	0.1374	[1.990323, 2.197432]
**SSO**	$1.606386\times 10^{-2}$	0.0569	[2.178347, 2.266937]
**$P_{\max}$ = 35 dBm**			
**Algorithm**	**Variance**	**CV**	**95% CI (Mean Objective)**
**CAOA**	$1.238377\times 10^{-1}$	0.4294	[0.7024033, 0.9481089]
**DO**	$2.286711\times 10^{-1}$	0.6553	[0.5710979, 0.9042849]
**NNA**	$2.623046\times 10^{-1}$	0.5096	[0.8327426, 1.1846810]
**SFO**	$1.679114\times 10^{-1}$	0.4646	[0.7457331, 1.0340040]
**ESFO**	$2.431900\times 10^{-1}$	0.2178	[2.0729660, 2.4185930]
**WSO**	$3.311786\times 10^{-1}$	0.5579	[0.8336210, 1.2377810]
**ECO**	$3.347758\times 10^{-1}$	0.3477	[1.457329, 1.859964]
**SSO**	$1.435254\times 10^{-1}$	0.2103	[1.664649, 1.928561]
**$P_{\max}$ = 40 dBm**			
**Algorithm**	**Variance**	**CV**	**95% CI (Mean Objective)**
**CAOA**	$1.385137\times 10^{-1}$	0.5895	[0.5059435, 0.7665772]
**DO**	$2.448687\times 10^{-2}$	0.8636	[0.1342849, 0.2420868]
**NNA**	$1.199483\times 10^{-1}$	0.7409	[0.3533313, 0.5947798]
**SFO**	$4.508111\times 10^{-2}$	0.6408	[0.2599604, 0.4087579]
**ESFO**	$3.591019\times 10^{-1}$	0.2703	[1.9812210, 2.4026960]
**WSO**	$8.173044\times 10^{-2}$	1.0112	[0.1933152, 0.3918948]
**ECO**	$3.611527\times 10^{-1}$	0.5858	[0.8186055, 1.236426]
**SSO**	$1.539777\times 10^{-1}$	0.4121	[0.8248328, 1.097589]
**$P_{\max}$ = 45 dBm**			
**Algorithm**	**Variance**	**CV**	**95% CI (Mean Objective)**
**CAOA**	$1.500356\times 10^{-1}$	0.7877	[0.3622092, 0.6305467]
**DO**	$1.134425\times 10^{-1}$	2.9851	[0.0423361, 0.2418184]
**NNA**	$1.656463\times 10^{-2}$	0.6794	[0.1477881, 0.2384155]
**SFO**	$3.639862\times 10^{-2}$	1.0817	[0.1159729, 0.2496978]
**ESFO**	$6.046327\times 10^{-1}$	0.3785	[1.7643330, 2.3052050]
**WSO**	$2.277383\times 10^{-2}$	1.3491	[0.0696177, 0.1731505]
**ECO**	$4.393845\times 10^{-1}$	1.0857	[0.3922489, 0.8604678]
**SSO**	$4.998676\times 10^{-2}$	0.4872	[0.3832307, 0.5389469]

**Table 5:** Wilcoxon signed-rank test results for different $P_{\max}$ levels (α = 0.05).

**Case 1:** $P_{\max}$**= 30 dBm**			
**Comparison**	***p*** **-Value**	**Signed-Rank**	**Significance**
**CAOA vs. ESFO**	$3.182\times 10^{-6}$	6	Significant
**DO vs. ESFO**	$5.706\times 10^{-4}$	65	Significant
**NNA vs. ESFO**	$2.564\times 10^{-2}$	124	Significant
**SFO vs. ESFO**	$1.477\times 10^{-4}$	48	Significant
**ESFO vs. WSO**	$3.182\times 10^{-6}$	459	Significant
**ESFO vs. ECO**	$1.197\times 10^{-3}$	75	Significant
**ESFO vs. SSO**	$2.957\times 10^{-3}$	88	Significant
**Case 2: $P_{\max}$ = 35 dBm**			
**Comparison**	***p*** **-Value**	**Signed-Rank**	**Significance**
**CAOA vs. ESFO**	$1.734\times 10^{-6}$	0	Significant
**DO vs. ESFO**	$2.879\times 10^{-6}$	5	Significant
**NNA vs. ESFO**	$9.711\times 10^{-5}$	43	Significant
**SFO vs. ESFO**	$1.734\times 10^{-6}$	0	Significant
**ESFO vs. WSO**	$4.729\times 10^{-6}$	455	Significant
**ESFO vs. ECO**	$4.196\times 10^{-4}$	61	Significant
**ESFO vs. SSO**	$8.307\times 10^{-4}$	70	Significant
**Case 3: $P_{\max}$ = 40 dBm**			
**Comparison**	***p*** **-Value**	**Signed-Rank**	**Significance**
**CAOA vs. ESFO**	$1.47\times 10^{-1}$	282	Not significant
**DO vs. ESFO**	$3.182\times 10^{-6}$	6	Significant
**NNA vs. ESFO**	$2.353\times 10^{-6}$	3	Significant
**SFO vs. ESFO**	$1.734\times 10^{-6}$	0	Significant
**ESFO vs. WSO**	$2.127\times 10^{-6}$	463	Significant
**ESFO vs. ECO**	$9.316\times 10^{-6}$	17	Significant
**ESFO vs. SSO**	$2.163\times 10^{-5}$	26	Significant
**Case 4: $P_{\max}$ = 45 dBm**			
**Comparison**	***p*** **-Value**	**Signed-Rank**	**Significance**
**CAOA vs. ESFO**	$6.339\times 10^{-6}$	13	Significant
**DO vs. ESFO**	$1.921\times 10^{-6}$	1	Significant
**NNA vs. ESFO**	$2.127\times 10^{-6}$	2	Significant
**SFO vs. ESFO**	$2.127\times 10^{-6}$	2	Significant
**ESFO vs. WSO**	$1.921\times 10^{-6}$	464	Significant
**ESFO vs. ECO**	$1.238\times 10^{-5}$	20	Significant
**ESFO vs. SSO**	$3.882\times 10^{-6}$	8	Significant

**Table 6:** Runtime and memory consumption comparison.

Algorithm	Elapsed Time (s)	MATLAB Memory Used (MB)
SFO	1.46457	3341
DO	1.5403	3298
CAOA	2.2217	3326
WSO	1.1406	3321
NNA	1.5186	3345
Proposed ESFO	1.3842	3353

**Table 7:** Performance comparison between SFO and ESFO on selected CEC 2017 benchmark functions under different dimensions.

**(i) Dimension = 2**						
	**F1**		**F2**		**F3**	
	**SFO**	**ESFO**	**SFO**	**ESFO**	**SFO**	**ESFO**
**Best**	500	500	800	800	1100	1100
**Average**	500	500	800	800	1100	1100
**Worst**	500	500	800	800	1100	1100
**SD**	0	0	0	0	0	0
**(ii) Dimension = 10**						
	**F1**		**F2**		**F3**	
	**SFO**	**ESFO**	**SFO**	**ESFO**	**SFO**	**ESFO**
**Best**	508.6591	507.9597	808.3378	804.9748	1101.625	1100.995
**Average**	520.4874	516.4368	822.7479	820.6951	1104.969	1104.907
**Worst**	533.8736	530.8437	841.7806	837.8083	1112.12	1110.945
**SD**	5.861489	5.117921	8.385096	8.187354	2.498186	2.290491
**(iii) Dimension = 30**						
	**F1**		**F2**		**F3**	
	**SFO**	**ESFO**	**SFO**	**ESFO**	**SFO**	**ESFO**
**Best**	659.6658	603.475	953.8448	866.3273	1205.09	1156.77
**Average**	725.9997	650.845	1022.94	939.4327	1317.633	1262.977
**Worst**	804.9961	719.9242	1108.06	1031.565	1534.308	1434.881
**SD**	35.73492	34.30399	36.93819	31.58044	67.63137	61.9013

## Data Availability

The datasets generated during and/or analyzed during the current study are available in the https://doi.org/10.5281/zenodo.19243208 repository.
